# Nodular Lymphocyte Predominant Hodgkin Lymphoma versus T-Cell/Histiocyte-Rich Large B-Cell Lymphoma: A Diagnostic Challenge

**DOI:** 10.1155/2014/956217

**Published:** 2014-07-07

**Authors:** Anton V. Rets, Susan R. S. Gottesman

**Affiliations:** Department of Pathology, State University of New York Downstate Medical Center, 450 Clarkson Avenue, Brooklyn, NY 11203, USA

## Abstract

Lymphomas with overlapping histological features of two distinct entities cause difficulty in classification. Their classification is of particular significance when the two alternatives require different treatment modalities. We present a diagnostically challenging case of a nodular lymphocyte predominant Hodgkin lymphoma (NLPHL) with features of T-cell/histiocyte-rich large B-cell lymphoma (THRLBCL). Our patient is a 39-year-old woman who presented with painless subclavicular and axillary lymphadenopathy. The biopsied lymph node showed diffuse architectural effacement and scattered large neoplastic cells with large irregular nuclei and prominent nucleoli. These cells were positive for CD20 and Bcl-6 and negative for CD15, CD30, IgD, and Bcl-2. The background cells were predominantly T lymphocytes, whereas B cells were markedly depleted. The lymph node was interpreted as NLPHL, consistent with THRLBCL-like variant. NLPHL, especially THRLBC-like variant, and de novo THRLBCL are characterized by significant morphologic and immunophenotypic overlap. Our case demonstrates a rare predominance of background T-cells in NLPHL and emphasizes the importance of thorough evaluation of multiple morphologic and immunophenotypic features as an essential approach for arriving at the correct diagnosis.

## 1. Introduction

Current classification of lymphomas is based on a complex of clinical, morphological, immunophenotypic, and molecular criteria. Such an approach provides an accurate classification of the majority of lymphomas. There are, however, cases which are difficult to fit into a distinct category. They demonstrate overlapping features between groups of lymphomas of different prognostic significance and treatment modalities. In such cases, making a correct diagnosis is a challenging task. Commonly these neoplasms show features of Hodgkin lymphomas (classical or nodular lymphocyte predominant (NLPHL)) and diffuse large B-cell lymphomas (DLBCL) or the latter and Burkitt lymphoma [1, 2]. For some, the dilemma cannot be resolved and a term “gray zone lymphoma” has been used to designate such cases. Here we present a diagnostically challenging case of a lymphoma with features of NLPHL and T-cell/histiocyte-rich large B-cell lymphoma (THRLBCL) with an attempt to distinguish between them and summarize their unique and overlapping diagnostic features.

## 2. Case Presentation

A 39-year-old obese woman presented with a painless 2 × 2 cm left subclavicular lymph node that she had had for 1-2 years. She denied any complaints, including B-symptoms, and her past medical and social histories were unremarkable. Physical examination revealed left supraclavicular and axillary lymphadenopathy and absence of hepatosplenomegaly. Peripheral blood was remarkable for mild normocytic normochromic anemia and mild neutropenia.

PET scan demonstrated bulky supraclavicular and axillary lymphadenopathy (SUV 8.01 and 11.89 resp.) with a neoplastic range hypermetabolic activity in the spleen and porta hepatis (SUV 10.65 and 12.1 resp.), as well as in the gastrohepatic and internal iliac lymph nodes. Bone marrow involvement was also suspected; however, no morphological evidence of involvement was demonstrated in the sample submitted. Cytogenetic analysis of the bone marrow revealed no abnormalities in karyotype by G banding with conventional trypsin-Giemsa technique and was negative for IGH/BCL1 and IGH/MYC translocation, as well as for rearrangements of BCL6, MALT1, ALK, and IGH genes by FISH.

**Figure 1:**
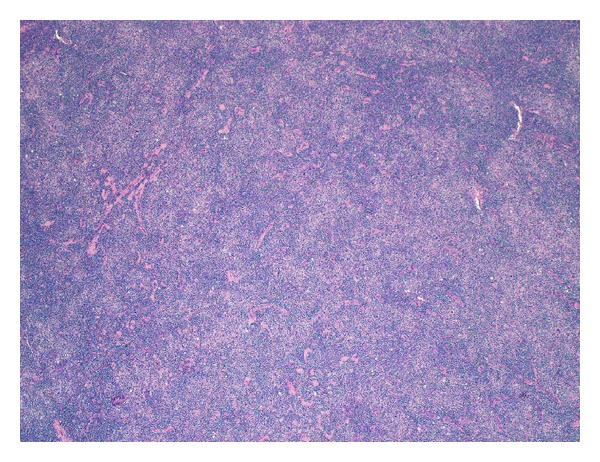
Biopsied lymph node architecture (hematoxylin and eosin stain, 40x). Vague nodules are present in the background of diffuse architectural effacement.

**Figure 2:**
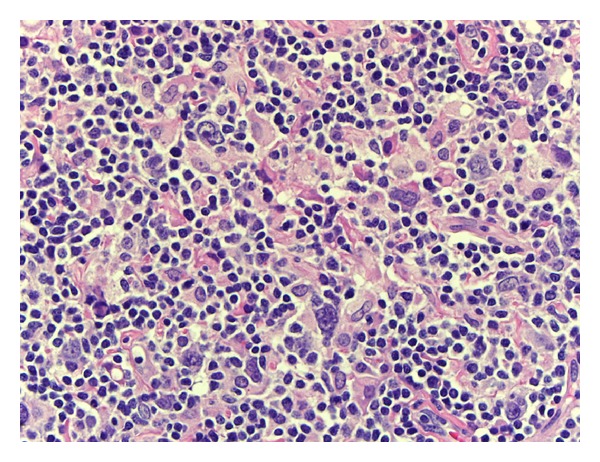
Neoplastic cells (hematoxylin and eosin stain, 400x). Neoplastic cells with one or multiple large pleomorphic nuclei and prominent nucleoli are admixed with histiocytes and benign lymphocytes.

The architecture of the biopsied supraclavicular lymph node was effaced and demonstrated moderate fibrosis, hyalinosis, and capsular thickening. The overall histologic appearance was predominantly diffuse, although small vague nodules could be appreciated (Figure 1). The neoplastic cells were large and scattered throughout the lymph node; they also formed loose clusters admixed with macrophages and small mature lymphocytes. These neoplastic cells had one or multiple, large, irregular, multilobulated nuclei with vesicular chromatin and prominent eosinophilic or amphophilic nucleoli (Figure 2). Numerous mitotic figures, including atypical ones, were present.

**Figure 3:**
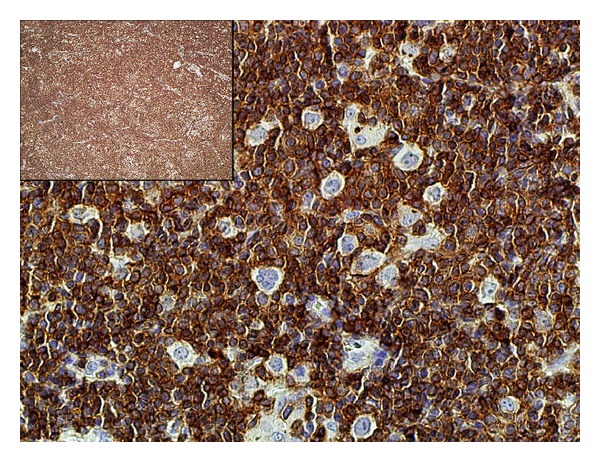
Background cells (CD3 immunostain, 400x; inlet, 40x). CD3-positive cells comprise the predominant background population.

**Figure 4:**
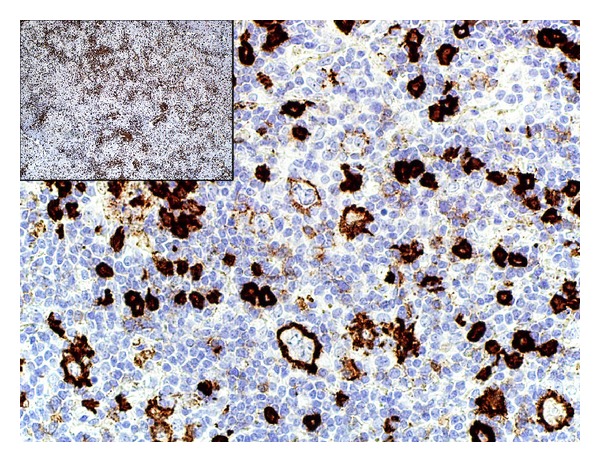
CD20-positive cells (CD20 immunostain, 40x; inlet, 40x). Highlighted B-cells are depleted and form loose clusters. Neoplastic cells strongly express CD20.

The neoplastic cells were positive for CD20 and Bcl-6 and negative for CD3, CD15, CD30, IgD, and Bcl-2. The background cells were predominantly T lymphocytes with CD20 : CD3 ratio of 1 : 10 (Figure 3). CD4+ population greatly outnumbered CD8+ cells (CD4 : CD8 ratio of 8 : 1). B-cells were markedly depleted and formed loose clusters (Figure 4). Besides a Bcl-6-positive B-cell population, these clusters contained a disrupted CD21-positive framework of follicular dendritic cells along with CD57 and PD1-positive T-cells, qualifying them as remnants of germinal centers. Occasional rosettes formed by PD1+ lymphocytes around the neoplastic cells were seen. The lymph node was interpreted as NLPHL, consistent with T-cell/histiocyte-rich large B-cell lymphoma-like variant (NLPHL THRLBCL-like variant). The clinical presentation corresponded with Stage IV disease by Ann Arbor system with Cotswolds modifications [3]. The patient was treated with 6 cycles of ABVD therapy and showed an appropriate initial response. The long-term therapeutic effect cannot be yet assessed.

## 3. Discussion

Our case illustrated a diagnostic struggle in differentiating between NLPHL and THRLBCL. The puzzling aspect of this case was a significant predominance of background T-cells over the B-cell population. This finding is exceptional in NLPHL, including its variants. NLPHL is usually characterized by background reactive B-cells predominating in the nodules, a feature of diagnostic importance, whereas, in a case of a THRLBCL, as the name suggests, T-cells are the main lymphoid population in the background. In fact, presence of neoplastic cells scattered in a T-cell and histiocyte-rich background devoid of small B-cells, observed in our case, is a prerequisite for the diagnosis of THRLBCL [4].

The diagnosis in this case, NLPHL THRLBCL-like variant, is an uncommon pattern of NLPHL. The morphologic resemblance of this neoplasm with THRLBCL poses a great diagnostic challenge. Distinguishing between these two entities is important because of the differences in their prognosis and treatment. Thus, NLPHL has an overall favorable prognosis, although worse clinical outcomes are expected in advanced stages. Historically, patients with NLPHL are treated like patients with classical Hodgkin lymphoma. Thus, limited field radiation therapy is the most common treatment for patients with an early stage disease [5, 6], whereas, in advanced-stage NLPHL, chemotherapy regimens may be preferred [7]. Unfortunately, up to 3–10% of cases progress to a DLBCL by 10 years [8]. THRLBCL, in contrast, is an aggressive neoplasm, prognostically comparable to DLBCL, not otherwise specified [9], and the chemotherapy modalities are significantly more intensive than those used for NLPHL [10, 11].

Both NLPHL and THRLBCL are characterized by the presence of scattered large neoplastic B-cells in a background of benign lymphocytes and macrophages. The neoplastic cells of the former, also known as LP cells, have scant cytoplasm and one folded or multilobated nuclei with prominent, mostly basophilic nucleoli. Although LP cells may have a phenotype overlapping with Hodgkin-Reed-Sternberg (HRS) cells, they are usually positive for CD45, CD20, CD79a, and Bcl-6, lack expression of CD15, and often are CD30-negative. IgD expression, seen in roughly one third of NLPHL, but not in our case, is more common in young men. In fact, IgD positivity is a useful argument against de novo THRBCL [12]. Large cells in THRLBCL may resemble LP and HRS cells but usually have more pronounced pleomorphism. Their immunoprofile is very similar to the former, with the exception of less common expression of Bcl-6 and more frequent expression of IRF4/MUM1. It is nearly impossible to establish a correct diagnosis based on the neoplastic cell morphology alone.

Histoarchitecture of the lymph node and the composition of the background cell populations provide the most reliable diagnostic features. The majority of NLPHLs demonstrate a nodular pattern. On rare occasions, diffuse architectural effacement may be seen, but even in that circumstance the background lymphocytes remain predominantly B-cells. Six distinct immunoarchitectural patterns of NLPHL have been described by Fan et al. 2003 [13]. They include NLPHL-classic B-cell-rich pattern (A), serpiginous pattern (B), pattern with prominent extranodular LP cells (C), T-cell-rich pattern (D), diffuse (THRLBCL-like) pattern (E), and diffuse pattern with a B-cell rich background (F). Patterns C–F may reflect an altered homing pattern of LP cells, with patterns D and E being associated with a T-cell-rich environment and progressive depletion of B-cells [14]. Presence of extranodular LP cells is believed to represent progression of nodular variant to diffuse pattern, with the latter being an independent predictor of relapse. Patients with variant NLPHL morphology, including THRLBCL-like, present in advanced stage (stage IIB and higher) as compared to cases with typical NLPHL pattern [14]. The THRLBCL-like pattern seen in our patient was also associated with an advanced stage at presentation, which reflects her long-standing history of undiagnosed NLPHL. Her delay in presenting for medical attention was most likely caused by her morbid obesity and absence of B-symptoms.

Regardless of the pattern, evidence of vague nodular structures can be demonstrated by immunohistochemistry which discloses a follicular dendritic cell meshwork containing B-cells, histiocytes, and CD4/CD57/PD1-positive T-cells. The PD1+ cells tend to form rosettes around the neoplastic cells, which can be used as a diagnostic feature of NLPHL, as in our case. Although it is frequently seen in nodular forms of NLPHL, diffuse patterns, including NLPHL THRLBCL-like, may even fail to exhibit rosette formation adding to the diagnostic difficulty [15–17].

In contrast, THRLBCL demonstrates a diffuse T-cell and histiocyte-rich infiltrate with few scattered tumor cells. Within the T-cell population, CD8+ cells usually outnumber the CD4+ subset. Small B-cells are present in minimal numbers; their prominence, even in a diffuse distribution, strongly argues against THRLBCL [18]. The presence of B-cell nodules associated with follicular dendritic cells also supports a diagnosis of NLPHL over THRLBCL [18]. A summary of diagnostic features of NLPHL and THRLBCL is presented in Table 1.

Despite morphological similarity, pathogenic relationship between NLPHL and THRLBCL remains controversial. The 2008 WHO Classification separates “de novo THCRLBCL” from “secondary THCRLBCL,” which may arise from NLPHL (also known as THRLBCL-like NLPHL), since they may represent distinct but morphologically and immunophenotypically similar entities [19]. A possible biologic link has been supported by molecular overlap between tumor cells of NLPHL (including NLPHL-THRLBCL-like) and de novo THRLBCL [17], a lack of disease specific pattern of gene expression, and similarities in their gene expression profile [20]. On the other hand, NLPHL demonstrates higher average number of genomic imbalances (10.8 versus 4.7 in THRLBCL) [21], which does not support the theory of a de novo THRLBCL being a form of progression of NLPHL. Indeed, NLPHL THRLBCL-like may represent a progression of a more typical NLPHL as patients present in advanced stage, as in our case.

## 4. Conclusion

NLPHL, especially THRLBC-like variant, and de novo THRLBCL are characterized by significant morphologic and immunophenotypic overlap. The composition of the background cell populations, usually a reliable feature to distinguish NLPHL from THRLBCL, may show a great degree of variability. Our case shows a rare predominance of the background T-cells in NLPHL and emphasizes the importance of thorough evaluation of multiple morphologic and immunophenotypic features as an essential approach for arriving at the correct diagnosis.

## Figures and Tables

**Table 1:** Diagnostic features of NLPHL, THRLBCL, and our case.

Criterion	NLPHL	TCHRLBCL	Our case
Clinical features			
Age	Any, most commonly between 30 and 50 years old	Middle age	39 years old
Gender predilection	Strong male predominance	Male predominance	Female
Presentation	Solitary peripheral lymphadenopathy	Advanced disease with lymphadenopathy, liver, spleen, and bone marrow involvement	Widespread peripheral lymphadenopathy with possible splenic, hepatic, and bone marrow involvement

Neoplastic cells			
Distribution	Nodular or diffuse and nodular	Diffuse; vague nodularity is rare	Diffuse and vaguely nodular
Bcl-6 expression	Present	Less common	Present
IRF4/MUM1 expression	Absent	May be present	Not performed
IgD expression	Present in approximately 1/3 cases	Absent	Absent
Average number of genomic imbalances	10.8	4.7	Not performed

Background cells			
Predominant lymphocyte population	B-cells	T-cells	T-cells
B lymphocytes distribution	Nodules	Nodules are absent	Vague nodules
CD4+ T lymphocytes	More prominent	Less prominent	Prominent
CD4+ versus CD8+ T lymphocytes	Mostly CD4+	Mostly CD8+	Mostly CD4+
Follicular center T lymphocytes (CD4+/CD57+/PD1+)	Present	Rare	Present
Follicular dendritic cell meshwork	Present	Absent	Present
PD1+ T lymphocyte rosettes around neoplastic cells	Present, may be absent in THRLBC-like variant	Absent	Occasional rosettes are present
